# Fertility-Sparing Strategies for Early-Stage Endometrial Cancer: Stepping towards Precision Medicine Based on the Molecular Fingerprint

**DOI:** 10.3390/ijms24010811

**Published:** 2023-01-03

**Authors:** Giuseppe Gullo, Gaspare Cucinella, Vito Chiantera, Miriam Dellino, Eliano Cascardi, Péter Török, Tünde Herman, Simone Garzon, Stefano Uccella, Antonio Simone Laganà

**Affiliations:** 1 Department of Obstetrics and Gynecology, Villa Sofia Cervello Hospital, University of Palermo, 90146 Palermo, Italy; 2 Unit of Gynecologic Oncology, ARNAS “Civico–Di Cristina–Benfratelli”, Department of Health Promotion, Mother and Child Care, Internal Medicine and Medical Specialties (PROMISE), University of Palermo, 90127 Palermo, Italy; 3 Department of Biomedical Sciences and Human Oncology, Obstetrics and Gynecology Section, Policlinic of Bari, University of Bari “Aldo Moro”, Piazza Aldo Moro, 70100 Bari, Italy; 4 Department of Medical Sciences, University of Turin, 10124 Turin, Italy; 5 Pathology Unit, FPO-IRCCS Candiolo Cancer Institute, Str. Provinciale 142 km 3.95, 10060 Candiolo, Italy; 6 Department of Obstetrics and Gynecology, Faculty of Medicine, University of Debrecen, 4032 Debrecen, Hungary; 7 Center of Assisted Reproduction, University of Debrecen, 4032 Debrecen, Hungary; 8 Department of Obstetrics and Gynecology, AOUI Verona, University of Verona, 37126 Verona, Italy

Endometrial cancer represents the fifth most common cancer in women, and the most common gynecological malignancy in developed countries [1]. Although endometrial cancer usually develops during the post-menopausal period, according to recent data, 15–25% of cases occur before menopause [2], when there may still be a desire of pregnancy. In premenopausal women, the most common clinical signs in the case of suspected endometrial cancer are abnormal uterine bleeding, intermenstrual bleeding, and to a lesser extent, pelvic pain [3]. These symptoms often prompt the patient to undergo a gynecological examination and ultrasound, which are of paramount importance for detecting endometrial thickness and altered eco-patterns, as well as other potential signs such as myometrial infiltration and cervical invasion [4]. Finally, an endometrial biopsy is crucial for obtaining the diagnosis of endometrial cancer and its grading. In this regard, accumulating evidence suggests that hysteroscopy-guided endometrial biopsy is associated with a higher diagnostic accuracy compared to blind endometrial sampling [5,6].

A fertility-sparing approach should be properly taken into account during the decision-making process regarding treatment, especially considering the clear detrimental psychological effect that comes from depriving women of their fertility potential due to an aggressive and radical approach [7]. On the one hand, women who are affected by endometrial cancer must receive the most appropriate treatments with curative intent [8]; on the other hand, these treatments should be modulated in order to preserve fertility [9]. The type of endometrial cancer is one of the most important parameters in deciding whether a woman can undergo fertility-sparing treatment. Indeed, women with endometrial cancer are usually affected by the type I endometrioid subtype, which is often estrogen dependent and low-grade, and has a better prognosis compared with type II. The type II endometrial cancer is, conversely, often estrogen-independent, of non-endometrioid histology, and myometrial-invasive [10]. From an epidemiological perspective, the main risk factors among women with the type I endometrioid subtype are obesity, anovulation, nulliparity, and hypoestrogenism [11]. Hypoestrogenism causes proliferation of the endometrium, which can lead to hyperplasia with or without atypia. Indeed, type I endometrial cancer is associated often with atypical hyperplasia as the pre-cancerous lesion; conversely, endometrial intraepithelial carcinoma, usually within the atrophic endometrium, is the precursor for type II non-endometrioid endometrial cancer [12].

In a real world scenario, patients with endometrial cancer during reproductive age who are candidates for a fertility-sparing approach have the stage IA, G1, low-grade endometrioid subtype [13]. However, myometrial [14], adnexal [15], and lymph-vascular space invasion must be excluded before offering a fertility-sparing approach [16]. Accumulating evidence suggests that hysteroscopic resection of the tumor, followed by hormonal treatment, could be acceptable in terms of oncological and reproductive outcomes [17]. Among the most frequently used pharmacological strategies for fertility preservation in this population, there are intrauterine devices containing levonorgestrel, alone or in combination with medroxyprogesterone acetate at a dose of 400–600 mg/daily, megestrol acetate at a dose of 160–320 mg/daily, or a gonadotropin-releasing hormone agonist [18].

At present, the new molecular classification (POLE-mutated/mismatch repair-deficient/no specific molecular profile/p53-abnormal) reported by the European Society of Gynaecological Oncology (ESGO), the European Society for Radiotherapy and Oncology (ESTRO), and the European Society of Pathology (ESP) consensus, published in 2021 [19], is expected to introduce new strategies for identifying patients at high- and low-risk of progression and relapse, and improving the selection of patients for a fertility-sparing approach [20]. This is of paramount importance, as the clinical pregnancy rate in women who undergo fertility-sparing treatments is about 35–60% with hormonal treatment alone, and approximately 70% after combined treatment based on hysteroscopic and hormonal therapy [21]; moreover, hysteroscopic resection followed by progestogen use is associated with a higher complete response rate, higher live birth rate, and lower recurrence rate than using oral progestogens alone [22]. The new potential available options, using the molecular classification, may also be applied in order to stratify women with atypical endometrial hyperplasia, a condition which can progress to endometrial cancer [23], as summarized in the review by Contreras et al. [24]. In addition, the molecular classification may also pave the way for increasing treatment in stage I G2 endometrioid endometrial cancer [25]. Indeed, the analysis of MMR, SPAG9, Ki67, and the Nrf2-survivin pathway provided results which were significantly associated with a good response to progestin therapy [26].

Based on this context, a robust systematic review by Tanos et al. [27], published in this Special Issue, suggested that PTEN and POLE alterations were found to be good prognostic factors of early-stage endometrial cancer, potentially allowing for fertility-sparing treatment, and confirming previous findings [28]. In addition, MSI, CTNNB1, and K-RAS alterations were found to be moderate prognostic factors of early-stage endometrial cancer, also allowing for fertility-sparing treatment; however, they were associated with a higher risk of recurrence compared with PTEN and POLE. Finally, PIK3CA, HER2, ARID1A, P53, L1CAM, and FGFR2 were found to be poor prognostic factors of early-stage endometrial cancer and, for this reason, fertility-sparing treatment in this population should be carefully considered, due to high risk of recurrence. Future strategies are aiming to use circulating tumor DNA as a “liquid biopsy” for the diagnosis of early-stage endometrial cancer [29,30]. This may represent an opportunity, especially for women of reproductive age in whom the diagnosis could be more difficult compared with post-menopausal women, due to nuanced symptoms and signs.

The decision regarding the utilization of a fertility-sparing approach should also take into account that several risk factors for endometrial cancer, including obesity and polycystic ovary syndrome, are also risk factors for infertility. Therefore, this may influence the need to use assisted reproduction technology in order to achieve pregnancy [31]. Interestingly, obese women have high levels of leptin and adiponectin due to higher production by adipose tissue and this, as summarized by Słabuszewska-Jóźwiak et al. [32], is known to play a key role in the most important signaling pathways of endometrial carcinogenesis. In this regard, the very interesting article by Olcha et al. [33] highlighted that tea polyphenols have proven antioxidative, anti-inflammatory, anti-obesogenic, and antidiabetic properties, and for these reasons may be considered as potential prophylaxis and pharmacotherapy support in endometrial cancer patients. Another new potential option that should be investigated in the future is D-Chiro-Inositol, which acts as an aromatase modulator, decreasing estrogen levels. As a result, it could be evaluated for use as a novel adjuvant strategy [34], and could perhaps provide similar beneficial effects to its stereoisomer, Myo-Inositol, which is used in menopausal transition [35].

Overall, the Special Issue “Endometrial Cancer in Reproductive Age: Fertility-Sparing Approach and Obstetric Outcomes” of the International Journal of Molecular Medicine offers novel insights regarding the management of this specific cancer population, and addresses research priorities that need to be investigated in the near future.

## Data Availability

Not applicable.
